# Tissue-Resident Type 2 Innate Lymphoid Cells Arrest Alveolarization in Bronchopulmonary Dysplasia

**DOI:** 10.1155/2020/8050186

**Published:** 2020-10-31

**Authors:** Lanlan Mi, Shaoxuan Zhu, Jiayu Cai, Suqing Xu, Zhengyang Xue, Hongyan Lu

**Affiliations:** Department of Pediatrics, The Affiliated Hospital of Jiangsu University, Zhenjiang 212001, China

## Abstract

Bronchopulmonary dysplasia (BPD) is a severe complication of the respiratory system associated with preterm birth. Type 2 innate lymphoid cells (ILC2s) play a major role in tissue homeostasis, inflammation, and wound healing. However, the role in BPD remains unclear. The present study showed that ILC2s, interleukin-4 (IL-4), IL-13, and anti-inflammatory (M2) macrophages increased significantly in BPD mice as compared to the control mice. Administration with recombinant mouse IL-33 amplified the above phenomena and aggravated the alveolar structural disorder and functional injury in mice subjected to BPD, and the opposite was true with anti-ST2 antibody. In addition, the depletion of ILC2s in BPD mice with anti-CD90.2 antibody substantially abolished the destructive effect on BPD. In the treatment of BPD with dexamethasone, the number of ILC2s and M2 macrophages and levels of IL-4 and IL-13 decreased with remission as compared to the control group. This study identified a major destructive role of the ILC2s in BPD that could be attenuated as a therapeutic strategy.

## 1. Introduction

Lung development is an important and complex process, which can be divided into five different stages: embryonic, pseudoglandular, canalicular, saccular, and alveolar. Aberrant lung development can lead to deleterious consequences for respiratory health such as bronchopulmonary dysplasia (BPD), a disease primarily affecting preterm neonates, which is now characterized by changes in pulmonary vascularization and alveolarization based on an arrest of lung development [1, 2].

The harmful effects of type 1-immunity-mediated inflammatory responses on lung development have been well confirmed, and the role of type 2 immune responses in postnatal lung development has also been studied in recent years. According to the study, from the beginning of the first breath, interleukin (IL)-33 increases rapidly and activates type 2 innate lymphoid cells (ILC2s) under the stimulation of mechanical stress and potential tissue damage of the lung, thus beginning to shape the type 2 immune environment of the lung [3].

Innate lymphoid cells (ILCs) are a group of non-B, non-T lymphocytes, which do not express antigen receptor during development, and participate in pathogen clearance, lymphoorganogenesis, and tissue remodeling. According to stereotyping the differentiated CD4+ T helper (Th) cells based on their cytokine production spectrum and the main transcription factors regulating their development, ILCs are divided into three major subtypes of type 1, 2, and 3.

ILC2s are the most abundant in pulmonary mucosa; although, they have also been detected in the liver, small intestine, bone marrow, and mediastinal and mesenteric lymph nodes [4]. Mirroring Th2 cells, ILC2s are activated by epithelial-derived cytokines, IL-25, IL-33, thymic stromal lymphopoietin (TSLP) through their cognate receptors, IL17RB, suppression of tumorigenicity 2 (ST2), and TSLPR, respectively. IL-33 is a member of the IL-1 family that is constitutively expressed in structural cells, such as type 2 pneumocytes in mice and lung epithelial cells in humans [5]. de Kleer et al. [6] demonstrated that the activation of the IL-33 pathway in the perinatal period promotes type 2 immunity of the developing lung, where in the alveolar epithelial cells (AECII) secrete IL-33 that regulates the type 2 immune responses by amplifying and activating ILC2s. Meanwhile, alveolar macrophages (AMs) can be detected as early as 10 days of gestation, and they are also continuously expressed in the process of fetal lung development [7], and their number will increase with alveolation [3, 8]. Moreover, increased ILC2s and M2 macrophages polarized by secreting IL-13 of ILC2s jointly stimulate the growth of AECII and promote the growth and repair of lung tissue after pneumonectomy [9]. In addition, ILC2s play a major role in a variety of lung diseases, such as helminth expulsion [10], airway inflammation [11], and atopic dermatitis [12]. However, whether ILC2s play a regulatory role in BPD, and the underlying mechanisms are yet to be clarified.

In the lung tissue of BPD newborn mice, the increased level of IL-33 participates in the development of BPD by regulating the inflammatory mediators, while the application of IL-33 inhibitor can protect the lung injury of newborn mice caused by hyperoxia [13]. The soluble receptor of IL-33, sST2, is used as an independent marker to predict the occurrence and development of BPD [14], indicating IL-33 plays an important role in BPD. Recent studies have also shown that IL-33 regulates the immune response of innate lymphocytes, especially the activation of ILC2s. ILC2s were initially identified as IL-5 and IL-13 producing cells [15]; they can also produce several other cytokines including IL-9 and IL-4. IL-33 also has the ability to promote the polarization of macrophages to M2 macrophages [16]. Importantly, the lungs of BPD mice exhibit predominantly type 2 immune responses, including increased Th2 chemokine levels [17], intense eosinophilic infiltration, Th2 hyperpolarization [18], the cytokines IL-4 and IL-13 [19], and M2 macrophages enrichment [20]. In conclusion, we speculated that ILC2s and M2 macrophages may have an important role in BPD by regulating type 2 immune responses.

To verify this hypothesis, we detected ILC2s, cytokines, and macrophages in lung tissue samples of LPS-induced BPD and assessed the effects of ILC2s on the immune response. Furthermore, the mechanism underlying ILC2s in the mediation of LPS-induced BPD was also explored.

## 2. Material and Methods

### 2.1. Mice

Wild-type C57BL/6 mice were purchased from the Animal Resources Centre of Jiangsu University (Jiangsu, China). All animals were maintained in the Jiangsu University Animal Facility and held under SPF conditions. For all studies, adult (8–12 weeks of age) mice were used in accordance with the animal care and use protocol approved by the Animal Resources Centre of Jiangsu University.

### 2.2. BPD Murine Model

The female adult mice mated 1 : 1 with the male at 5 : 00 p.m. in the afternoon, and vaginal plugs were observed at 8 : 00 a.m. on the next morning. The day that vaginal plug was found was recorded as gestational age day 0.5. On day 16.5 of gestation (term: 21 days), pregnant mice were prepared for receiving intra-amniotic injections. The pregnant mice were randomly assigned to the saline control group and lipopolysaccharide (LPS) (Sigma, USA) group. The saline control group received 5 *μ*L of normal saline per amniotic sac, while the LPS group received 1 *μ*g of LPS diluted to 5 *μ*L with normal saline per amniotic sac. This study uses the method of Liu et al. and the method description partly reproduces their wording [21]. The day when the pups were born was counted as postnatal day 1 (P1). Neonatal mice from different litters were euthanized for lungs on P1, P3, P7, P14, and P21.

### 2.3. Hematoxylin-Eosin (H&E) Staining

Lung tissue from mice was washed with PBS and fixed in 4% paraformaldehyde (Biosharp, China), then paraffin-embedded. Paraffin sections were dewaxed with xylene solution and placed in an alcohol solution for 2 min. After normal dehydration, the sections were transparently processed and sealed. Then, the tissues were stained with hematoxylin and eosin. Finally, the staining results were observed under an optical microscope. Images were captured at 200x magnification.

### 2.4. Flow Cytometry

Lungs were isolated, minced, ground by 100-*μ*m cell strainers, and passed through a 70-*μ*m cell strainer. After red cell lysis using ACK (Leagene, China), the cell suspension obtained from a single mouse was washed with phosphate-buffered saline (PBS), and the cells counted. For FACS analysis, the single-cell suspensions were stained with antibodies to CD11b-FITC (101206), F4/80-APC/Cyanine7 (123118), CD86-PE (E-AB-F0994D), CD206-PE/Cyanine7 (E-AB-F1135D), CD90.2-PE (E-AB-F10940) [3, 22], and Sca-1-APC (108112), as well as antibodies to Lineage-FITC (133302) [4] (all purchased from eBioscience or Biolegend, USA). Cells were analyzed on a flow cytometer (Beckman CytoFLEX, USA).

### 2.5. Enzyme-Linked Immunosorbent Assay (ELISA)

IL-33, IL-4, and IL-13 levels in lung were assayed using an ELISA kit (MultiSciences Biotech Co., Ltd., China). ELISA was performed according to the manufacturer's protocol.

### 2.6. Real-Time Quantitative Reverse Transcription PCR (qRT-PCR)

Total RNA was isolated from the lung by TRIzol reagent (Vazyme Biotech Co., Ltd, China), and reverse transcription was then performed according to the instructions of HiScript Q RT SuperMix for qPCR (Vazyme Biotech Co., Ltd, China). Diluted complementary deoxyribose nucleic acid (cDNA), primers, premix, and ultrapure water were mixed into a 10-*μ*L reaction system. Specific cDNA amplification was performed using an ABI 7500 FAST real-time PCR machine with ChamQ SYBR qPCR Master Mix (Vazyme Biotech Co., Ltd, China), according to the manufacturer's protocol. Primers used in this study were as follows: IL-13 and IL-4 (MQP027416, MQP032451, GeneCopoeia, USA).

### 2.7. IL-33 and Anti-ST2 Antibody Administration

For IL-33 treatment, neonatal (P7) mice were administered 0.2 *μ*g mouse recombinant IL-33 (CG73, BioLegend, USA) [23] intraperitoneally every alternate day and euthanized for lungs on P14. For IL-33 functional depletion, neonatal (P7) mice from different litters were administered a 0.5 *μ*g mouse anti-ST2 antibody [24] (MAB10041-SP, BioLegend, USA) intraperitoneally every alternate day and euthanized for lungs on P14. The control mice received mice IgG (BE0093, Bioxcell, USA).

### 2.8. ILC2s Depletion

For ILC2s depletion, neonatal (P7) mice from different litters were given anti-CD90.2 antibody (50 *μ*g per mouse; E-AB-F10940, BioLegend, USA) intraperitoneally [25] every other day and were euthanized for lungs on P14. The control group received mice IgG.

### 2.9. Dexamethasone (DEX) Treatment

To study the role of DEX (Sigma, USA), neonatal (P7) mice were administered the drug (2.5 mg/kg) intraperitoneally [26] every day. Control mice received only PBS. The animals from different litters were euthanized at P14 to harvest the lungs.

### 2.10. Statistical Analyses

Statistical tests included unpaired, two-tailed *t*-test using Welch correction for unequal variances and one-way ANOVA with Tukey multiple comparison test. Statistical analysis was performed using Prism (Version 8; GraphPad, USA). Results are expressed as the mean ± SEM. *P* < 0.05 was considered statistically significant.

## 3. Results

### 3.1. IL-33 and Tissue-Resident ILC2s in Lung Increased Markedly in BPD Mice

**Figure 1:**
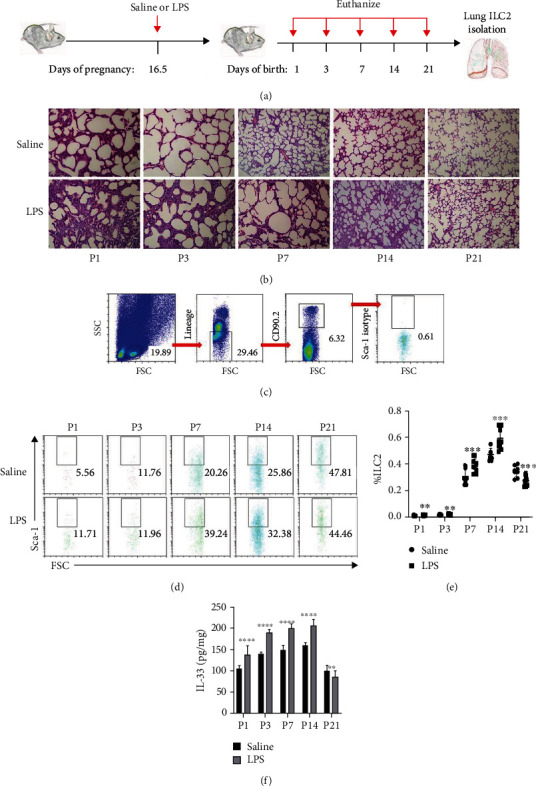
IL-33 and tissue-resident ILC2s in lung increased markedly in BPD mice. (a) BPD C57BL/6 mice model was established with lipopolysaccharide (LPS) using pregnant mice at 16.5 days of gestation. Neonatal mice were euthanized for lungs on P1, P3, P7, P14, and P21. (b) Representative hematoxylin-eosin-stained sections of the lung from C57BL/6 mice treated with saline or LPS. Original magnification, ×200. (c) Representative FACS analysis shows the gating strategy to identify Lin^−^CD90.2^+^Sca-1^+^ILC2 in mouse lung. (d) Representative results of ILC2s detected by flow cytometry on different postnatal days in saline (control) and LPS (BPD) groups. (e) Percentage of ILC2 in the lung on different postnatal days in saline and LPS groups. (f) The expression of IL-33 in the lung on different postnatal days in saline and LPS groups. Data are represented as mean ± SD. *n* = 10. The LPS group was compared with the saline group at the same time point, and ^∗∗^ indicates *P* < 0.01, ^∗∗∗^ indicates *P* < 0.001, and ^∗∗∗∗^ indicates *P* < 0.0001.

To determine whether IL-33 and ILC2s modulate the occurrence and development of BPD, BPD mice were established and assessed for IL-33 and ILC2s (Figure 1(a)). In the control group, with the increase in age, the lung tissue developed gradually, the alveolar structure was regular, and the alveolar wall became thinner, while in the BPD group, the alveolar structure was disordered, the alveolar fusion became larger, the number decreased, the structure simplified, and the alveolar wall gradually thickened, which was similar to the pathological changes in human BPD, indicating the successful establishment of the model (Figure 1(b)). ILC2s increased in BPD mice as compared to that in control mice and were significantly improved in mice during 7 and 14 days after birth (Figures 1(c)–1(e)). The level of IL-33 was high after birth and gradually increased with the increase in age up to 14 days, and then decreased on day 21 after birth. The level of IL-33 in the BPD group was higher than that in the control group until 14 days after birth, while the opposite was true on day 21 after birth (Figure 1(f)). Taken together, these results indicated that IL-33 and ILC2s play a major role in the development of BPD.

### 3.2. Cytokine Levels and Macrophages in the Lung Also Increased Markedly in BPD Mice

**Figure 2:**
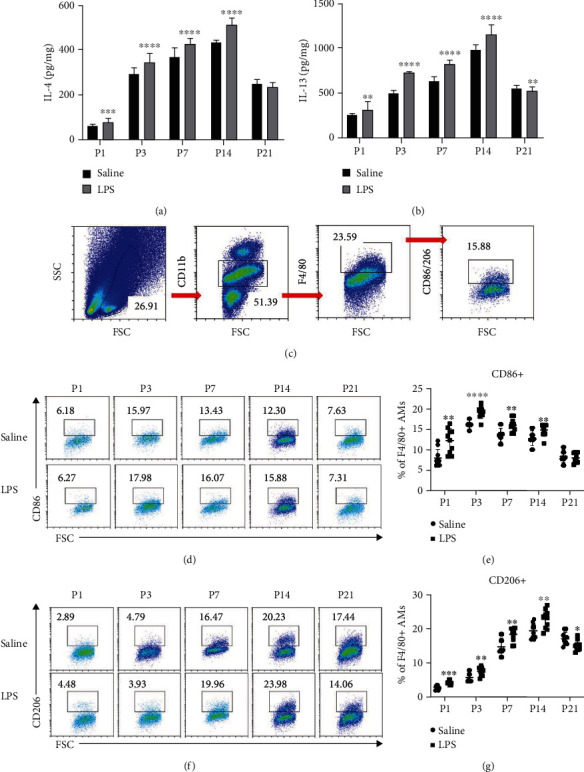
Cytokine levels and macrophages in lung increased markedly in BPD mice. (a, b) The expression of IL-4 and IL-13 in the lung on different postnatal days in saline and LPS groups, respectively, both *P* < 0.05*vs*. saline. (c) Representative FACS analysis showing the gating strategy to identify CD11b^+^F4/80^+^CD86^+^M1 and CD11b^+^F4/80^+^CD206^+^M2 macrophages in mouse lung. (d, e) Representative results of M1 and M2 macrophages detected by flow cytometry on different postnatal days in saline and LPS groups, respectively. (f, g) Percentage of M1 and M2 macrophages in the lung on different postnatal days in saline and LPS groups, respectively. Data are represented as mean ± SD. *n* = 10. The LPS group was compared with the saline group at the same time point, and ^∗^ indicates *P* < 0.05, ^∗∗^ indicates *P* < 0.01, ^∗∗∗^ indicates *P* < 0.001, and ^∗∗∗∗^ indicates *P* < 0.0001.

The detection of cytokines in lung tissues of mice showed that the expression levels of IL-4 and IL-13 in the BPD group were significantly higher than those in the control group. The levels of IL-4 and IL-13 increased first and then decreased, and the peak value was at the end of rapid alveolation (day 14 after birth). Also, the level of IL-13 was distinctly higher than that of IL-4. In addition, the expression levels of IL-4 and IL-13 were higher in BPD mice (Figures 2(a) and 2(b)) than the control group. Since both cytokines can polarize macrophages to M2 type, we detected the change in the occurrence of macrophages and the development of BPD. Flow cytometric analysis of CD11b^+^F4/80^+^CD206^+^M2 macrophages in lung confirmed that the number of M2 macrophages, increased first and then decreased after birth and peaked on the day 14 after birth. Also, the number of M2 macrophages in BPD was significantly higher than those in the control group before 14 days after birth (Figures 2(c), 2(e), and 2(g)). Flow cytometric analysis of CD11b^+^F4/80^+^CD86^+^M1 macrophages showed that the number of M1 macrophages increased at 3 days after birth and then decreased. Moreover, the number of M1 macrophages in BPD was higher than that in the control group before 14 days after birth (Figures 2(c), 2(d), and 2(f)), which was generally consistent with the altered degree of inflammation of BPD.

### 3.3. IL-33 Enhanced the Number of ILC2s Dramatically in the Lung of BPD Mice

**Figure 3:**
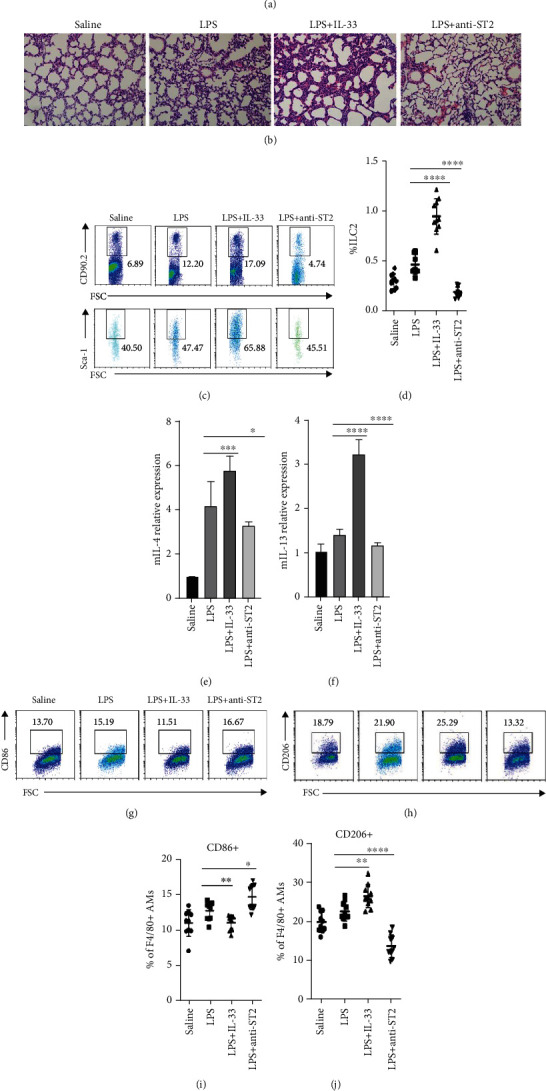
IL-33 enhanced the number of ILC2s dramatically in the lung of BPD mice. (a) C57BL/6 BPD mice were treated with mouse recombinant IL-33, anti-ST2 antibody, or PBS every alternate day from day 7 after birth. Neonatal mice were euthanized for lungs on P14. (b) Representative hematoxylin-eosin-stained sections of the lung from C57BL/6 mice treated with saline, LPS+vehicle (IgG), LPS+IL-33, and LPS+anti-ST2. Original magnification, ×200. (c) Representative results of ILC2 detected by flow cytometry on different postnatal days in saline, LPS+vehicle, LPS+IL-33, and LPS+anti-ST2 groups. (d) Percentage of ILC2s in the lung on different postnatal days in saline, LPS+vehicle, LPS+IL-33, and LPS+anti-ST2 groups. (e, f) Relative expression of IL-4 and IL-13 in the lung in saline, LPS+vehicle, LPS+IL-33, and LPS+anti-ST2 groups, respectively. (g, h) Representative results of M1 and M2 macrophages detected by flow cytometry in saline, LPS+vehicle, LPS+IL-33, and LPS+anti-ST2 groups, respectively. (i, j) Percentage of M1 and M2 macrophages in the lung in saline, LPS+vehicle, LPS+IL-33, and LPS+anti-ST2 groups, respectively. Data are represented as mean ± SD. *n* = 10. ^∗^ indicates for *P* < 0.05, ^∗∗^ indicates *P* < 0.01, ^∗∗∗^ indicates *P* < 0.001, and ^∗∗∗∗^ indicates *P* < 0.0001.

To study the role of IL-33 in BPD, we treated C57BL/6 mice with mouse recombinant IL-33 (0.5 *μ*g/mouse/day intraperitoneally) or anti-sST2 antibody (1 *μ*g/mouse/day intraperitoneally) every alternate day from day 7 after birth and sacrificed mice on day 14 (Figure 3(a)). In BPD mice treated with IL-33, the structure of alveoli was disordered, the fusion of alveoli was larger, the number was reduced, the structure was simplified, the alveoli wall was thickened, and the inflammatory cells were increased, while the opposite was true in BPD mice treated with anti-ST2 antibody (Figure 3(b)). In addition, ILC2s increased notably in BPD mice that interfered with IL-33, while the effect of intervention with anti-ST2 antibody was reversed (Figures 3(c) and 3(d)). Therefore, the current data showed that IL-33 markedly aggravated lung injury and inflammation in BPD mice and ILC2s. Furthermore, the evaluation of the lung tissue of mice showed that the expression levels of IL-4 and IL-13 in BPD mice with IL-33 were significantly higher than those in the BPD group, and the phenomenon was opposite in BPD mice with anti-ST2 antibody (Figures 3(e) and 3(f)). Similarly, M2 macrophages in BPD mice with IL-33 were markedly higher, while those in BPD mice with anti-ST2 antibody were opposite (Figures 3(h) and 3(j)). However, the changes in M1 macrophages in BPD mice with IL-33 and anti-ST2 antibody were opposite to that of M2 macrophages (Figures 3(g) and 3(i)).

### 3.4. Depletion of ILC2s Attenuate BPD

**Figure 4:**
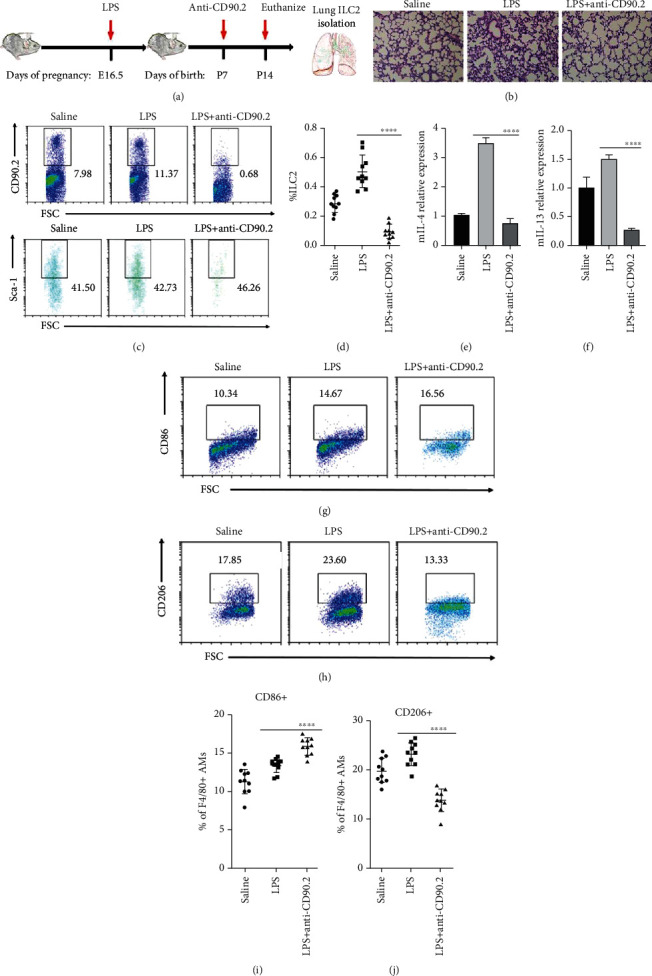
Depletion of ILC2s attenuates BPD. (a) C57BL/6 BPD mice were treated with mouse anti-CD90.2 antibody or IgG2b every alternate day from day 7 day after birth. Neonatal mice were euthanized for lungs on P14. (b) Representative hematoxylin-eosin-stained sections of the lung from C57BL/6 mice treated with saline, LPS, LPS+vehicle (IgG), and LPS+anti-CD90.2. Original magnification, ×200. (c) Representative results of ILC2s detected by flow cytometry on different postnatal days in saline, LPS+vehicle, and LPS+anti-CD90.2 groups. (d) Percentage of ILC2 in the lung on different postnatal days in saline, LPS+vehicle, and LPS+anti-CD90.2 groups. (e, f) Relative expression of IL-4 and IL-13 in the lung in saline, LPS+vehicle, and LPS+anti-CD90.2 groups, respectively. (g, h) Representative results of M1 and M2 macrophages detected by flow cytometry in saline, LPS+vehicle and LPS+anti-CD90.2, groups, respectively. (i, j) Percentage of M1 and M2 macrophages in lung in saline, LPS+vehicle, and LPS+anti-CD90.2 groups, respectively. Data are represented as mean ± SD. *n* = 10. ^∗∗∗∗^ indicates *P* < 0.0001.

The potential contribution of ILC2s to IL-33-mediated destruction of BPD was assessed in C57BL/6 mice. To verify the role of ILC2s in BPD, we treated the mice with anti-CD90.2 antibody (50 *μ*g/neonatal mouse/day intraperitoneally) every alternate day from day 7 day after the birth; the animals were sacrificed on day 14 (Figure 4(a)). Flow cytometric analysis of Lin^−^CD90.2^+^Sca-1^+^ ILC2s in the lung confirmed significant depletion of ILC2s in mice with anti-CD90.2 antibody treatment as compared to IgG-treated control mice (Figures 4(c) and 4(d)). With the decrease in ILC2s, the alveolar structure tended to be regular, the number of alveoli increased, and the alveolar wall became thinner (Figure 4(b)). Hence, the depletion of ILC2s by the administration of anti-CD90.2 antibody had a discernible effect on lung injury and lung inflammation in BPD. Interestingly, the lung tissue of mice showed that the expression levels of IL-4 and IL-13 in BPD mice with anti-CD90.2 antibody decreased significantly (Figures 4(e) and 4(f)). Similarly, M2 macrophages in BPD mice with anti-CD90.2 antibody decreased markedly (Figures 4(h) and 4(j)), while the changes in M1 macrophages in BPD mice with anti-CD90.2 antibody were opposite (Figures 4(g) and 4(i)).

### 3.5. DEX Reduces the Inflammation and Injury in BPD by Regulating ILC2s

**Figure 5:**
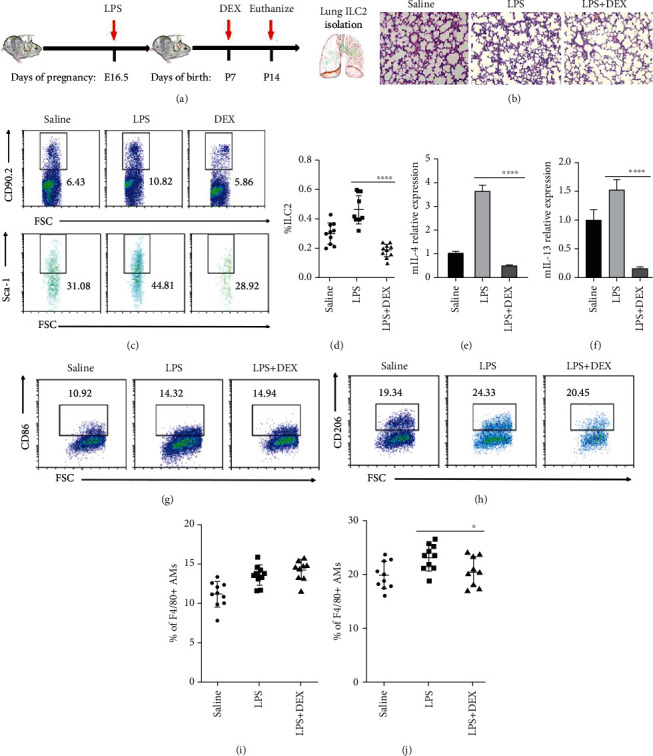
Downregulation of ILC2s involved in the regulation of DEX to alleviate BPD. (a) C57BL/6 BPD mice were treated with DEX or PBS daily from day 7 after birth. Neonatal mice were euthanized for lungs on P14. (b) Representative hematoxylin-eosin-stained sections of the lung from C57BL/6 mice treated with saline, LPS+vehicle (PBS) and LPS+DEX. Original magnification, ×200. (c) Representative results of ILC2s detected by flow cytometry on different postnatal days in saline, LPS+vehicle, and LPS+DEX groups. (d) Percentage of ILC2s in the lung on different postnatal days in saline, LPS+vehicle, and LPS+DEX groups. (e, f) Relative expression of IL-4 and IL-13 in the lung in saline, LPS+vehicle, and LPS+DEX groups, respectively. (g, h) Representative results of M1 and M2 macrophages detected by flow cytometry on different postnatal days in saline, LPS+vehicle, and LPS+DEX groups, respectively. (i) Percentage of M1 macrophages in the lung on different postnatal days in saline, LPS+vehicle, and LPS+DEX groups. (j) Percentage of M2 macrophages in the lung on different postnatal days in saline. Data are represented as mean ± SD. *n* = 10. ^∗^ indicates *P* < 0.05; ^∗∗∗∗^ indicates *P* < 0.0001.

DEX is a common reliever used for BPD in clinics and is also known to inhibit type 2 cytokine expression by blood ILC2s stimulated with IL-33 [27, 28]. In order to investigate whether DEX affects ILC2s in BPD, we treated C57BL/6 mice with DEX (2.5 mg/kg intraperitoneally) daily from day 7 after birth and sacrificed the animals on day 14 (Figure 5(a)). After DEX treatment, the lung injury and pneumonia of BPD mice were relieved, the alveolar structure normalized, and the alveolar septum became thinner (Figure 5(b)). In addition, the number of ILC2s detected by flow cytometry and the expression of IL-4 and IL-13 decreased (Figures 5(c)–5(f)). The number of M2 macrophages detected by flow cytometry decreased significantly (Figures 5(h) and 5(j)), while that of M1 macrophages changed slightly (Figures 5(g) and 5(i)). Thus, it can be deduced that DEX reduces the inflammation and injury in BPD by regulating ILC2s.

## 4. Discussion

BPD is a chronic lung disease of primarily premature infants, which is characterized by impaired alveolar development. About 50% of very low birth weight infants are exposed to infection or inflammation caused by chorioamnionitis before birth, which impairs lung development, resulting in BPD [29]. Although it has been found that intrauterine infection can lead to lung maturation and even decrease of neonatal respiratory distress syndrome, the model of BPD induced by chorioamnionitis is still one of the common models of BPD [30, 31]. Besides, Liu et al. [21] successfully induced the BPD model by injecting LPS into amniotic cavity of pregnant rats on day 16.5. According to this kind of method, we established a BPD model caused by intrauterine infection. The results of histopathology showed that in the LPS group, the structure of alveoli tended to be disordered, the fusion of alveoli became larger, the number of alveoli decreased, the lung structure simplified, and the alveoli wall gradually thickened, which were similar to the pathological changes of human BPD, indicating that the BPD modeling was successful.

To determine whether ILC2s play a regulatory role in BPD, we established the BPD mouse. The results provided evidence that the number of ILC2s in the lung of BPD mice increased first and then decreased, peaking at the end of rapid alveolation (P14), and were significantly higher than that in the control group before P14, similar to the expression level of IL-33. Furthermore, the expression levels of IL-4 and IL-13 in the BPD group also increased than that in the control group, and the level of IL-13 was much higher than that of IL-4. Since both cytokines can polarize macrophages to the M2 type, we speculated that M2 macrophages are also involved in BPD. Herein, we found that the number of M2 macrophages in the alveoli of BPD mice was higher than that of the control group, and this trend was consistent with that of ILC2s. Therefore, we speculated that ILC2s activated by IL-33 play a regulatory role in the occurrence and development of BPD; also, IL-4, IL-13, and M2 macrophages are involved.

To verify the role of IL-33 in BPD, we injected recombinant IL-33 intraperitoneally to increase the level of IL-33 in BPD mice and knocked down the receptor of IL-33 by anti-ST2 antibody to block the effect of the cytokine. The results showed that the alveolar volume expanded, the alveolar septum thickened, and the severity of pneumonia increased in BPD mice treated with IL-33. Therefore, we speculated that the increase of IL-33 level aggravates the condition of BPD. Strikingly, the above phenomena were reversed after the application of the anti-ST2 antibody. Previous studies supported the hypothesis that IL-33 mediates deterioration in BPD [14, 32]. IL-33 contributes significantly to sepsis-induced inflammation in the lung, early inflammation-associated lung injury, and systemic inflammatory response in the early phase of sepsis by upregulating the level of ILC2s [33]. A recent study demonstrated that attenuating the ILC2s response activated by IL-33 suppresses the allergic lung inflammation [34]. However, whether the expansion of ILC2s is responsible for IL-33-mediated damage in BPD has not been addressed before. In this study, we found that not only the ILC2s in the lungs of BPD mice increased significantly as compared to the control group but also increased more significantly after IL-33 intervention. Surprisingly, ILC2s notably reduced in BPD mice with anti-ST2 antibody. The expression levels of IL-4 and IL-13 in BPD mice with IL-33 were significantly higher than that in BPD group, and the phenomenon was opposite to that of anti-ST2 antibody. Moreover, M2 macrophages significantly increased in the lung of IL-33-treated BPD mice while notably decreased in the lung of anti-ST2 antibody-treated BPD mice, suggesting that IL-33 can promote the polarization of M2 macrophages in mice. These results provided further evidence for the hypothesis that ILC2s activated by IL-33 play a regulatory role in BPD and IL-4, IL-13, and M2 macrophages.

However, whether ILC2s are beneficial for the occurrence and development of BPD is yet to be elucidated. In current studies [25, 35], anti-CD90.2 antibodies have often been used for depletion of ILC2s, although the possibility of simultaneous knockdown of other cell types cannot be ruled out. In the study of ischemia-reperfusion injury, ILC2s were depleted by anti-CD90.2 antibodies to study structural and functional injury of the kidney [25]. In atherosclerosis studies, anti-CD90.2 antibodies have been used to deplete ILC2s to study changes in plaque size and macrophage infiltration [35]. Thus, we exhausted ILC2s in BPD mice via anti-CD90.2 antibody intraperitoneally. The structure of alveoli became regular, the number of alveoli increased markedly, the walls of alveoli became thin, and pneumonia was alleviated in BPD mice after depleting ILC2s. These results showed that the depletion of ILC2s significantly eliminated the destructive effect of IL-33 in BPD mice, clearly indicating that ILC2s constitute a major pathway in IL-33-mediated destruction in this model.

In addition, the expression levels of IL-4 and IL-13 decreased significantly in BPD mice with ILC2s depletion as compared to the control group. A previous study identified that natural RV infection in severely premature young children elicits airway secretion of Th2 (IL-4 and IL-13) cytokines that are associated with a history of BPD [19]. Zhao et al. [36] demonstrated that ILC2s were recruited into the fibrotic lung through the IL-33/ST2 pathway and promoted the activation of fibroblasts through the production of IL-13 to promote pulmonary fibrosis. In chronic anaphylactic pneumonia, alveolar epithelial cells have been confirmed to undergo epithelial-mesenchymal transition (EMT) to form activated fibroblasts, and the proportion of EMT cells is related to the expression of *IL-13* mRNA [37]. As a major regulator of fibrosis, IL-13 enhances the EMT process [38]. ILC2s are the major sources of IL-13 in vivo, and hence, might be involved in the process of EMT. Another study has shown that purified ILC2s reinfusion aggravates lung inflammation and interstitial cell deposition; also, the expression of TGF-*β*1 and other EMT inducible factors is increased [16]. In this study, when the expression level of IL-13 increased, the alveolar structure was disordered, and the alveolar septum was thickened. These phenomena were reversed when the expression level of IL-13 decreased significantly, indicating that IL-13 plays a critical role in the regulation of BPD by ILC2s. However, further studies are needed to determine the role of IL-13 in BPD.

Importantly, IL-4 and IL-13 promote the polarization of AMs to M2 macrophage phenotype, and the data showed that the trend of M2 macrophages in BPD was consistent with these two cytokines. Based on the defect of ILC2s, the number of M2 macrophages decreased by a wide margin, indicating that ILC2s regulate the polarization of macrophages in vivo. Previous findings showed that the coculture of ILC2s with AMs induced the expression of M2 macrophage-related genes in the in vitro setting [39]. A clinical study showed that newborn infants from mothers with histological chorioamnionitis (one of the important causes of BPD) had lower levels of classically-activated macrophages (M1) and higher levels of alternatively-activated macrophages (M2) in blood samples and tracheal aspirates than newborn infants from healthy mothers [20]. These findings indicated that ILC2s activated by IL-33 play a destructive role in BPD, partially via modulation of lung macrophage phenotype. The results also showed that the number of M2 macrophages was dominant from P7 to P14, while that of M1 macrophages was higher from P1 to P3. In addition, the number of M1 macrophages in BPD group was significantly higher than that in the control group, indicating that M1 macrophages may play an important role in promoting the inflammation of BPD in the early stage of birth. However, Willis et al. [40] found that promoting the M2 macrophage polarization improves lung function and lung injury in the hyperoxia-induced BPD model. A previous study also showed that hyperoxia differentially contributed to macrophage polarization by enhancing LPS-induced M1 and inhibiting IL-4-induced M2 phenotype in vitro [41]; these contrasting results could be explained by various models and in vivo and in vitro differences or in different periods.

In view of the application of DEX in the late stage of BPD in clinic [42], we used DEX to intervene the mice model in the late stage of this disease, in order to explore whether the mechanism of DEX is related to ILC2s. The present study provided evidence that DEX could blunt lung inflammation and improve lung injury in BPD by downregulating IL-13 and alveolar macrophages via ILC2s. Findings showed that activated ILC2s are partially attenuated by corticosteroids, and DEX induces apoptotic cell death [28, 43]. However, the mechanism of DEX in BPD is not addressed clearly, with respect to its effect through ILC2s. In this study, the lung inflammation was reduced, and the lung injury was improved in DEX-treated BPD mice by decreasing ILC2s. Moreover, with the downregulation of ILC2s, the expression levels of IL-4 and IL-13 decreased. However, the application of DEX-reduced M2 macrophages had no significant effect on M1 macrophages, suggesting that other cells and mechanisms might contribute to DEX-mediated protection in BPD.

**Figure 6:**
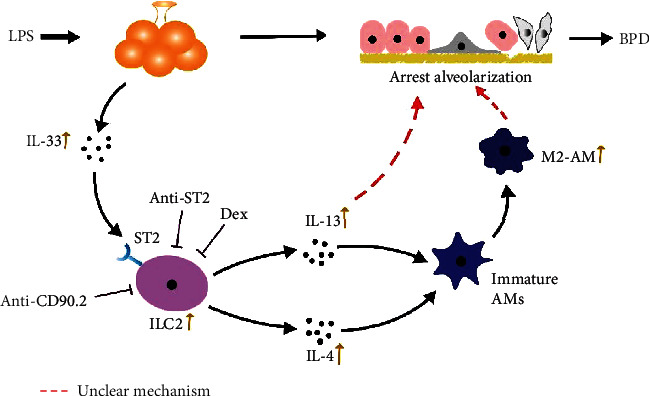
A schematic representation of the mechanism of ILC2s on regulating the development of BPD.

In conclusion, these data suggest that ILC2s play an important regulatory role in the formation and development of BPD. After being activated by IL-33, ILC2s secrete a large number of IL-13 and IL-4, which promote the polarization of M2 macrophages, and play a role through IL-13 and M2 macrophages (Figure 6). Anti-ST2 antibody and anti-CD90.2 antibody can reverse the above results, and DEX has the same effect (Figure 6). However, the detailed molecular mechanism of IL-13 and M2 macrophages needs to be further investigated (Figure 6).

There are still some limitations to this study. Although we found that ILC2s play a destructive role in BPD, the detailed molecular mechanism remains to be elucidated. In addition, these in vivo results should be further validated in vitro. Although we found that ILC2s can act through IL-13 and M2 macrophages, we have no idea whether ILC2s can directly act on AECII. All these limitations should be addressed in the following study.

## 5. Conclusion

In summary, a major finding of this study is that IL-33-elicited ILC2s play a critical role in the destruction in BPD, which is shown for the first time. Also, the negative effect of ILC2s is exerted via IL-13 directly or M2 macrophages indirectly. Furthermore, the protective effect of DEX in BPD is effectuated at least partially through the regulation of ILC2s. Thus, the present study suggested a novel therapeutic approach of inhibiting the innate immune response of ILC2s for BPD, and further research is needed.

## Data Availability

All data generated or analyzed during this study are included in this published article.
